# Prostate specific membrane antigen (PSMA) PET/CT for radiation planning and response assessment in intracranial high-grade gliomas

**DOI:** 10.1093/noajnl/vdag217

**Published:** 2026-08-17

**Authors:** Rituraj Upadhyay, Pavnesh Kumar, Divya Yadav, Bingfeng Tang, Erika Bello-Pardo, Hamid Mohtashami, Pawan Singh, Pierre Giglio, Kyle C Wu, James Brad Elder, Sasha Beyer, Raj Singh, Raju R Raval, Evan M Thomas, Chi Zhang, Jana Ivanidze, Joshua D Palmer

**Affiliations:** Department of Radiation Oncology, Moffitt Cancer Center, Tampa, Florida, USA; Department of Radiation Oncology, The Ohio State University Wexner Medical Center, Columbus, Ohio, USA; Department of Radiation Oncology, The Ohio State University Wexner Medical Center, Columbus, Ohio, USA; Department of Diagnostic Imaging, Moffitt Cancer Center, Tampa, Florida, USA; Department of Diagnostic Radiology and Nuclear Medicine, The Ohio State University Wexner Medical Center, Columbus, Ohio, USA; Ohio State University College of Medicine, Columbus, Ohio, USA; Division of Neuro-Oncology, Department of Neurology, The Ohio State University Wexner Medical Center, Columbus, Ohio, USA; Division of Neuro-Oncology, Department of Neurology, The Ohio State University Wexner Medical Center, Columbus, Ohio, USA; Division of Neuro-Oncology, Department of Neurology, The Ohio State University Wexner Medical Center, Columbus, Ohio, USA; Department of Neurosurgery, The Ohio State University Wexner Medical Center, Columbus, Ohio, USA; Department of Neurosurgery, The Ohio State University Wexner Medical Center, Columbus, Ohio, USA; Department of Radiation Oncology, The Ohio State University Wexner Medical Center, Columbus, Ohio, USA; Department of Radiation Oncology, The Ohio State University Wexner Medical Center, Columbus, Ohio, USA; Department of Radiation Oncology, The Ohio State University Wexner Medical Center, Columbus, Ohio, USA; Renaissance Institute of Precision Oncology & Radiosurgery, Orlando, Florida, USA; Department of Radiation Oncology, Mayo Clinic, Rochester, Minnesota, USA; Department of Radiology, Weill-Cornell Medicine, New York, New York, USA; Department of Radiation Oncology, The Ohio State University Wexner Medical Center, Columbus, Ohio, USA

**Keywords:** glioblastoma, high-grade gliomas, PSMA PET/CT, radiation necrosis, tumor control

## Abstract

**Background:**

Prostate-specific membrane antigen (PSMA) expression has been demonstrated in the tumor microenvironment of high-grade gliomas (HGG). Here, we assessed the utility of PSMA-positron emission tomography (PET) imaging in patients with HGG for radiation therapy (RT) planning and in differentiating tumor progression from post-treatment changes.

**Methods:**

Patients with HGG (WHO grade 3-4) were included in our prospective PET imaging registry. Analysis was stratified into two cohorts based on clinical indication for PET—Cohort A: RT planning; and Cohort B: evaluation of suspected recurrence. PSMA avidity was quantified using standardized uptake value (SUV) measurements including absolute maximum SUV(SUVmax), and SUV ratio referencing right parotid (SUVRp), and superior sagittal sinus (SUVRsss).

**Results:**

Twenty-seven patients were included (Cohort A, *N* = 19; Cohort B, *N* = 8). In Cohort A, all MRI T1 contrast-enhancing lesions were PET avid; with PSMA-avid volume exceeding MRI T1 contrast-enhancing volume in 4 patients (20%). The respective mean lesion SUVmax, SUVRp, and SUVRsss for patients with PSMA avid disease (*n* = 17) vs those without (*n* = 2) were 7.2 vs 1.5 (*P* = .002); 0.6 vs 0.2 (*P* = .008); and 6.5 vs 1.8 (*P* = .008). Post-RT mean SUVmax decreased by 25.8% (6.2 to 4.6, *P* = .005). In Cohort B, PSMA PET accurately identified pathologically confirmed tumor progression (*n* = 6, mean SUVmax 7.0) and necrosis (*n* = 1, SUVmax 1.5) in 88% patients.

**Conclusions:**

PSMA PET avidity, particularly SUVmax, is higher in glioblastoma multiforme (GBM) patients with residual tumor or tumor progression. These findings suggest a potential role for PSMA PET in tumor delineation, aiding in comprehensive coverage of tumor volumes for radiation planning and response assessment to differentiate tumor progression and post-treatment changes.

## Lay Summary

Glioblastoma is an aggressive brain cancer, and it can be difficult to tell whether changes seen on scans after radiation treatment represent tumor growth or treatment-related effects. The authors of this study wanted to evaluate whether a specialized imaging test called PSMA PET could help define tumor boundaries for radiation planning and distinguish tumor progression from treatment-related changes. To do this, they performed PSMA PET scans in 27 patients with high-grade gliomas, including patients undergoing radiation planning and patients being evaluated for possible tumor recurrence. They found that tumors showed higher PSMA PET signal than surrounding tissue, that PSMA PET sometimes identified tumors beyond what was visible on standard MRI, and that it correctly distinguished tumor progression from treatment-related changes in most patients.

Key Points

- PSMA PET SUVmax is elevated in high-grade glioma patients with residual tumor and improves target delineation.
- PET SUV of treated tumor demonstrated considerable decrease following chemoradiation.
- PSMA PET accurately identified tumor progression and necrosis.

Importance of the Study

High-grade gliomas have a poor prognosis despite standard-of-care multi-modal treatment. There is a strong need for better predictive and prognostic imaging biomarkers. Prostate-specific membrane antigen (PSMA) positron emission tomography (PET) is FDA-approved and widely used in the diagnosis and treatment of prostate cancers. PSMA expression has been demonstrated in the tumor microenvironment of high-grade gliomas (HGG). We performed this study to assess the utility of PSMA-PET in radiation treatment planning, response assessment, and differentiating true progression from post-treatment changes in patients with HGG. In this cohort of 27 patients with HGG, we were able to demonstrate an increased PSMA PET avidity, particularly lesion SUVmax, in glioblastoma multiforme (GBM) patients with residual tumor or tumor progression; PET identified additional areas of tumor beyond areas of MRI defined tumor in 20% patients, aiding in adequate coverage with radiation therapy (RT); PET standardized uptake value (SUV) decreased markedly post chemoRT; and PSMA PET accurately identified tumor progression and necrosis with pathological correlation in 88% patients.

Intracranial high-grade gliomas (HGG), which include WHO Grade 3 and 4 gliomas, have poor prognosis with a median survival of 15-20 months, despite multi-modality treatment. Among these, glioblastoma multiforme (GBM) is the most common and aggressive primary malignant brain tumor.^1^ Standard-of-care treatment comprises maximal safe resection of the tumor followed by radiation therapy (RT) with concurrent and adjuvant chemotherapy.^2^^,^^3^ Contrast-enhanced magnetic resonance imaging (MRI) is the current standard to evaluate tumor extent and treatment response. Conventional MRI has several limitations in assessing post-operative residual disease and post-radiation response due to overlapping MRI characteristics of tumor and post-treatment changes. Several studies have documented early tumor progression between surgical resection and initiation of RT in glioblastoma patients.^4^^,^^5^ In addition, contrast enhancement following radiation can mimic tumor progression, commonly referred to as pseudoprogression.^6^^,^^7^ Advanced imaging techniques such as perfusion MRI, MR spectroscopy, and 18F-Fluorodeoxyglucose Positron Emission Tomography (FDG PET) scans have been incorporated into clinical practice to aid in differentiating viable tumor from post-treatment changes but have limited sensitivity and specificity.^8^ There remains a strong clinical need for predictive and prognostic imaging biomarkers as well as novel treatment modalities in the management of high-grade gliomas (HGG) to improve treatment outcomes.

Prostate specific membrane antigen (PSMA) is overexpressed on the surface of prostate cancer cells and has demonstrated excellent clinical utility for prostate cancers, allowing both imaging with 18F- and 68Ga-PSMA PET, as well as radionuclide therapy using 177Lu-PSMA.^9^^,^^10^ Preliminary studies have also demonstrated PSMA overexpression in GBM and high-grade gliomas.^11^^,^^12^ Early analyses of resected GBM specimens have shown high and heterogeneous PSMA expression on immunohistochemistry (IHC), either diffuse expression in both tumor stroma and the endothelium or primarily endothelial expression.^11^^,^^13^ PSMA expression has also been found to be a negative prognostic marker. Feasibility of in vivo PET imaging with PSMA-targeted PET in primary brain neoplasms has also been demonstrated recently.^12^^,^^14^ Several case reports and small series have reported the utility of 68Ga-PSMA and 18F-PSMA-based imaging in evaluation of brain tumors, especially in differentiating tumor progression from post-radiation changes.^15^ PSMA-targeted PET imaging thus represents a promising noninvasive approach for evaluating residual tumor burden after surgery and/or RT in patients with HGG. This could further set the stage for a paradigm-shifting theranostics approach to treatment of these aggressive tumors. However, the diagnostic accuracy and clinical utility of PSMA PET in gliomas remain unclear, limiting its clinical implementation. We undertook this study to evaluate the utility of PSMA-PET/CT in detecting postoperative residual tumor to help in RT planning and response assessment after RT, as well as its role in differentiating tumor progression from post-treatment changes in patients with suspected radiographic recurrence of HGG.

## Methods

### Patient Selection

This was a prospective, single-center PET imaging registry study approved by the Institutional Review Board. PSMA PET/CT was offered to all patients with HGG after surgery for RT planning and at follow-up for suspected recurrence; and consecutive patients who agreed to get a PET scan were enrolled on our registry. Patients enrolled between January 2022 and December 2024 were included in this study. Other study inclusion criteria were: (1) pathologically confirmed grade 3 or grade 4 gliomas; (2) PSMA PET/CT performed after surgical resection or biopsy either prior to RT or at follow-up after suspected GBM recurrence; and (3) patients that received definitive intent RT [biologically equivalent dose (BED) > 50 Gy]. Patients who did not complete their planned course of RT or did not have any follow-up imaging available for review were excluded from this study.

### Radiation Simulation and Treatment Planning

All patients had a contrast enhanced MRI at baseline within 2 weeks of CT simulation for radiation planning. Patients were simulated supine with a thermoplastic mask and treated on a linear accelerator using volumetric modulated arc therapy (VMAT) with daily kV cone-beam CT image guidance. Treatment target volumes were delineated on fused T1 contrast-enhanced and T2 FLAIR MRI sequences based on RTOG guidelines. The prescribed radiation dose varied based on fractionation, with most patients receiving 60 Gy in 30 fractions. T2 FLAIR abnormality with margin received 46 Gy in 23 fractions, while contrast-enhancing tumor with margin received a sequential boost to a total dose of 60 Gy in 30 fractions. Alternatively, older patients or patients with poor performance status received a hypofractionated regimen of 52.5 Gy in 15 fractions to the contrast-enhancing tumor and 40 Gy to the T2 FLAIR volume with margin. Patients received dose escalation to PSMA PET-avid tumor to 60 Gy in 15 fractions using simultaneously integrated boost when clinically appropriate. All patients received concurrent temozolomide 75 mg/m^2^ daily. Typical radiation dose for patients receiving re-irradiation after prior RT for lower-grade gliomas was 35 Gy in 10 daily fractions over 2 weeks.

### PSMA PET-CT

For PET imaging, 68Ga-PSMA-11 (Gozetotide) with an activity of 3-5 mCi was injected and approximately 60 minutes following the radiotracer injection the patient was positioned on the Siemens Biograph mCT PET/CT scanner to obtain a low-resolution non-contrast CT from the top of the head through the mid-femurs for use in attenuation correction and anatomic correlation. PET emission scans of this anatomic region (vertex to thigh) were subsequently acquired. While the brain was the primary focus, a whole-body survey was performed per the standard institutional oncology PET protocol. Axial, sagittal, coronal PET, CT, fused PET/CT, and maximal intensity projection PET images were reconstructed per standard guidelines and were available for clinical interpretation and RT planning. PSMA avidity was quantitatively analyzed using standardized uptake value (SUV) measurements, including maximum SUV of the lesion (SUVmax), SUV ratio referencing right parotid (SUVRp), and SUV ratio referencing superior sagittal sinus (SUVRsss) based on prior work.^16^^,^^17^

### Follow-up

Patients were divided into 2 groups based on the timing of PET relative to their radiation treatment course—Cohort A undergoing PSMA PET following resection and prior to RT, and Cohort B undergoing PET at follow-up for suspected recurrence. Patients were followed every 2-3 months after completing RT with contrast-enhanced MRI Brain. Patients continued toadjuvant temozolomide after completing RT as per standard guidelines. A follow-up PSMA PET/CT was also obtained to evaluate for progression vs post-treatment changes. Patients with tumor progression underwent salvage therapy, including surgical resection, re-irradiation, or other modalities, based on disease characteristics and clinical performance status. The last clinic visit, imaging, or date of contact was used for censoring patients alive at the time of analysis. Patient and tumor-level data for each course were extracted. Follow-up details were extracted from the electronic medical record (EMR), and individual MRI images and/or pathology were reviewed to determine tumor progression vs radiation necrosis (RN). Pathological correlation was used to determine tumor progression or RN wherever available, otherwise, serial MRI changes were considered as reference.

### Statistical Analysis

Our primary endpoint was the feasibility of PET-guided differentiation of tumor and post-treatment changes. Summary statistics for patient characteristics and SUVs are presented as both mean and standard deviation (SD) as well as median and interquartile ranges (IQR). Intergroup comparisons were performed using Mann-Whitney test for independent samples or Wilcoxon matched-pairs signed rank test for paired samples. Kaplan-Meier method was used for survival analysis. Progression-free survival (PFS) and Overall Survival (OS) were defined from the date of diagnosis of HGG to the last follow-up or progression (PFS), or death (OS), from any cause, with censoring of patients who were lost to follow-up. OS was also separately reported from the date of PSMA PET scan and from the end of RT to the date of death or last follow-up. Cox proportional hazards model was used for analysis of prognostic factors affecting OS and PFS. Logistic regression analyses were summarized using hazard ratios (HR) and their 95% confidence intervals (CI). A *P*-value of 0.05 or less was considered statistically significant. Statistical tests were based on a 2-sided significance level. All statistical analyses were performed using R v4.2.2 (R Core Team) and SPSS v31.0 (Armonk, IBM Corp, NY).

## Results

From January 2022 to December 2024, a total of 27 patients with HGG underwent PSMA PET-CT, of which 19 patients underwent PET prior to baseline RT (Cohort-A), while 8 patients underwent PET at the time of suspected recurrence (Cohort-B). Baseline patient characteristics and treatment details are described in Table 1.

**Table 1.** Patient characteristics

	Cohort A *N* (%)	Cohort B *N* (%)
Number of patients	19	8
Age (y): median (IQR)	66.0 (60.8-73.2)	61.3 (50.4-67.5)
Sex: Male	14 (74%)	2 (25%)
WHO Grade		
III	1 (5%)	0
IV	18 (95%)	8 (100%)
IDH wildtype	18 (95%)	7 (88%)
MGMT methylation	6 (32%)	5 (63%)
Surgery		
Gross total resection (GTR)	8 (42%)	4 (50%)
STR	8 (42%)	4 (50%)
Biopsy only	3 (16%)	0
Radiation dose		
60 Gy in 30 fractions (Median)	11 (58%)	8 (100%)
52.5 Gy in 15 fractions	6 (31%)	0
35 Gy in 10 fractions	2 (11%)	0
PET guided dose escalation	2 (11%)	N/A
Concurrent Temozolomide	19 (100%)	8 (100%)

### Cohort-A

A total of 19 patients underwent PET-guided radiation treatment, using PET avidity to define target volumes and assist in radiation planning. The median age of patients was 66 years (IQR 60.8-73.2 years). Of these, 14 were males (74%), 18 were WHO grade 4 (95%), and 6 (32%) had O6-Methylguanine-Methyltransferase (MGMT) gene methylated tumors. Eight patients had gross-total resection (42%), 8 had subtotal resection (42%) and 3 underwent biopsy only (16%). All patients underwent adjuvant RT with concurrent Temozolomide, with a median dose of 60 Gy (60 Gy/30 fractions = 11, 52.5 Gy/15 fractions = 6, 35 Gy/10 fractions = 2). Two patients had a prior history of grade 2 and grade 3 astrocytoma respectively, treated with surgery and adjuvant radiation 2-5 years prior to current HGG diagnosis, and subsequently received PET-guided reirradiation 35 Gy in 10 fractions. The median times from surgery to PET imaging was 5 weeks and surgery to RT was 6 weeks (range 3-7 weeks).

### Imaging Findings

Following surgical intervention, PSMA PET avid lesions with elevated SUV compared to background brain activity were identified in 17 patients (89%), while MRI reported residual disease in 14 patients (74%), equivocal findings in 2 patients (10%), and no residual tumor in 3 patients (16%). One patient with PSMA avid disease had no evidence of residual or recurrent tumor on MRI, while additional areas of PSMA avidity beyond T1 contrast-enhancing and T2 FLAIR hyperintense areas on MRI were identified in 4 patients (21%), aiding in comprehensive coverage of tumor volumes during radiation planning. All MRI-enhancing tumors were PET avid as well. The mean (±SD) lesion SUVmax, SUVRp, and SUVRsss for patients with PSMA-avid disease (*n* = 17) vs those without (*n* = 2) was 7.23 ± 2.34 vs 1.53 ± 0.39 (*P* = .002, Mann Whitney test); 0.57 ± 0.20 vs 0.15 ± 0.16 (*P* = .008); and 6.47 ± 2.78 vs 1.78 ± 1.69 (*P* = .008), respectively. Figure 1A-C demonstrate the boxplots comparing respective SUVmax, SUVRp, and SUVRsss in patients with and without PSMA-avid disease prior to RT. PET-guided dose escalation was also performed in 2 patients, who were treated with 40 Gy in 15 fractions to the tumor and T2 FLAIR signal, while the PET-avid areas of disease were escalated to 60 Gy in 15 fractions.

**Figure 1.**
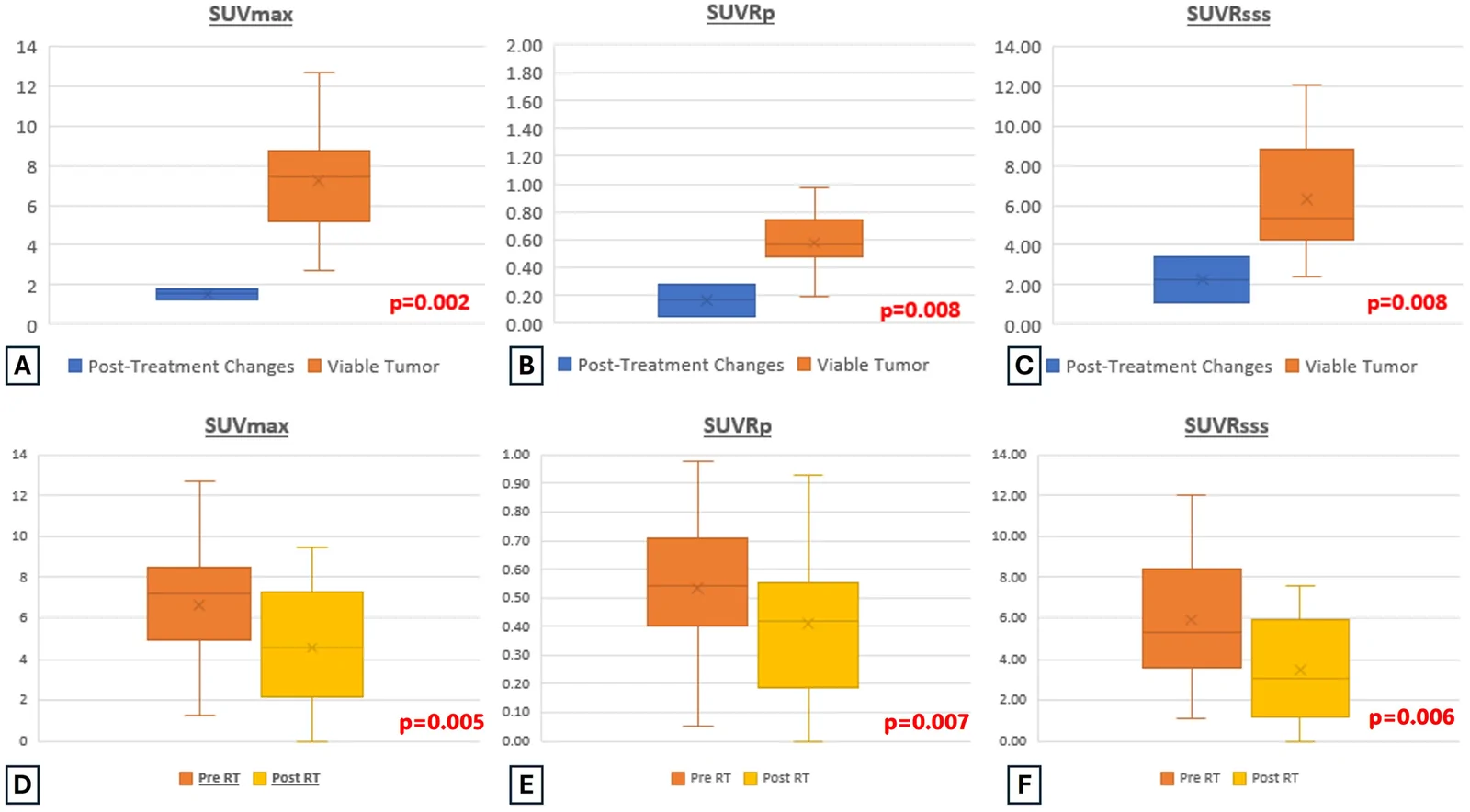
Boxplot demonstrating imaging findings on PSMA PET in patients with PET-avid tumor vs those without (A: SUVmax, B: SUV ratio referencing right parotid [SUVRp], C: SUV ratio referencing superior sagittal sinus [SUVRsss]) (Mann-Whitney *P*-values); and boxplots demonstrating imaging findings on PSMA PET before and after RT in patients with PET avid tumor at baseline (D: SUVmax, E: SUVRp, F: SUVRsss) (Wilcoxon matched-pairs signed rank *P*-values).

Figures 2A, 3, and 4 demonstrate examples of representative cases demonstrating the feasibility and benefits of PSMA PET imaging in these patients. Figure 2A shows corresponding PSMA PET avidity in a patient with rapid tumor progression postoperatively. Figure 3 shows a case where PSMA PET was able to confirm a gross total resection (GTR), despite MRI being equivocal, and subsequent imaging a year later showed PSMA-avid area corresponding to a suspicious marginal area of recurrence on MRI. Figure 4 shows a patient with varying degrees of PSMA PET avidity in the postoperative bed after a subtotal resection, and we were able to simultaneously boost the PSMA-avid residual tumor to a higher dose of 60 Gy in 15 fractions.

**Figure 2.**
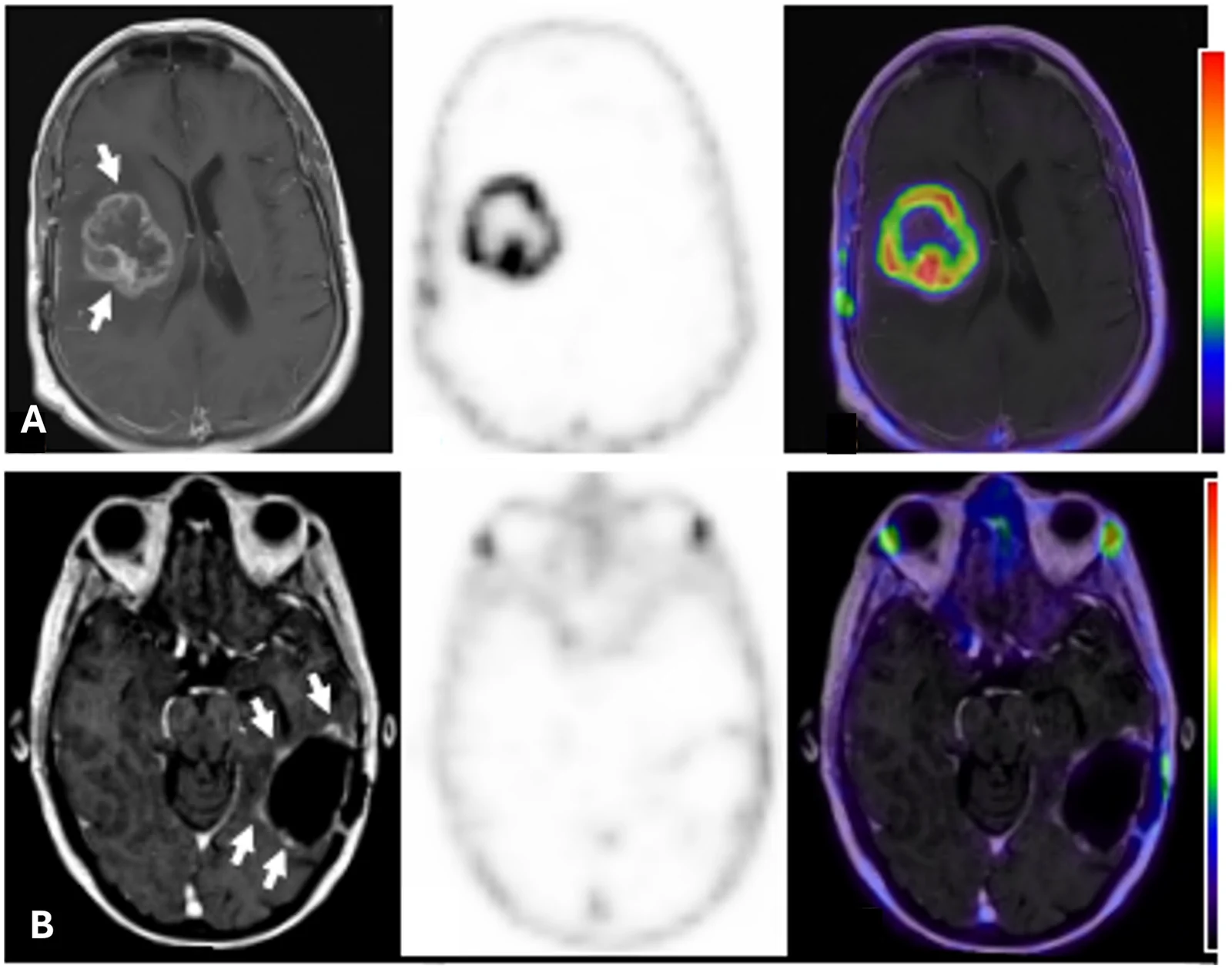
Top panel (A): A 68-year-old woman who presented with headaches and was found t have a right corona radiata mass. She underwent partial resection and pathology demonstrated glioblastoma. About 4 weeks after initial resection, MRI demonstrated increased nodular enhancement deep to resection cavity suspicious for recurrence, with corresponding PSMA PET scan showing moderate avidity (SUV 7). Bottom panel (B): PSMA PET at follow-up supporting post-radiation necrosis. This is a 51-year-old who initially was diagnosed with left temporal GBM and underwent resection and chemoradiation. His follow-up imaging (E) showed new peri-cavitary enhancement at about 14 months after diagnosis but only minimal PSMA avidity (SUV max 1.5). This patient underwent a surgical re-excision and was found to have only gliosis and no progressive tumor. Color Bar SUV 0-5.

**Figure 3.**
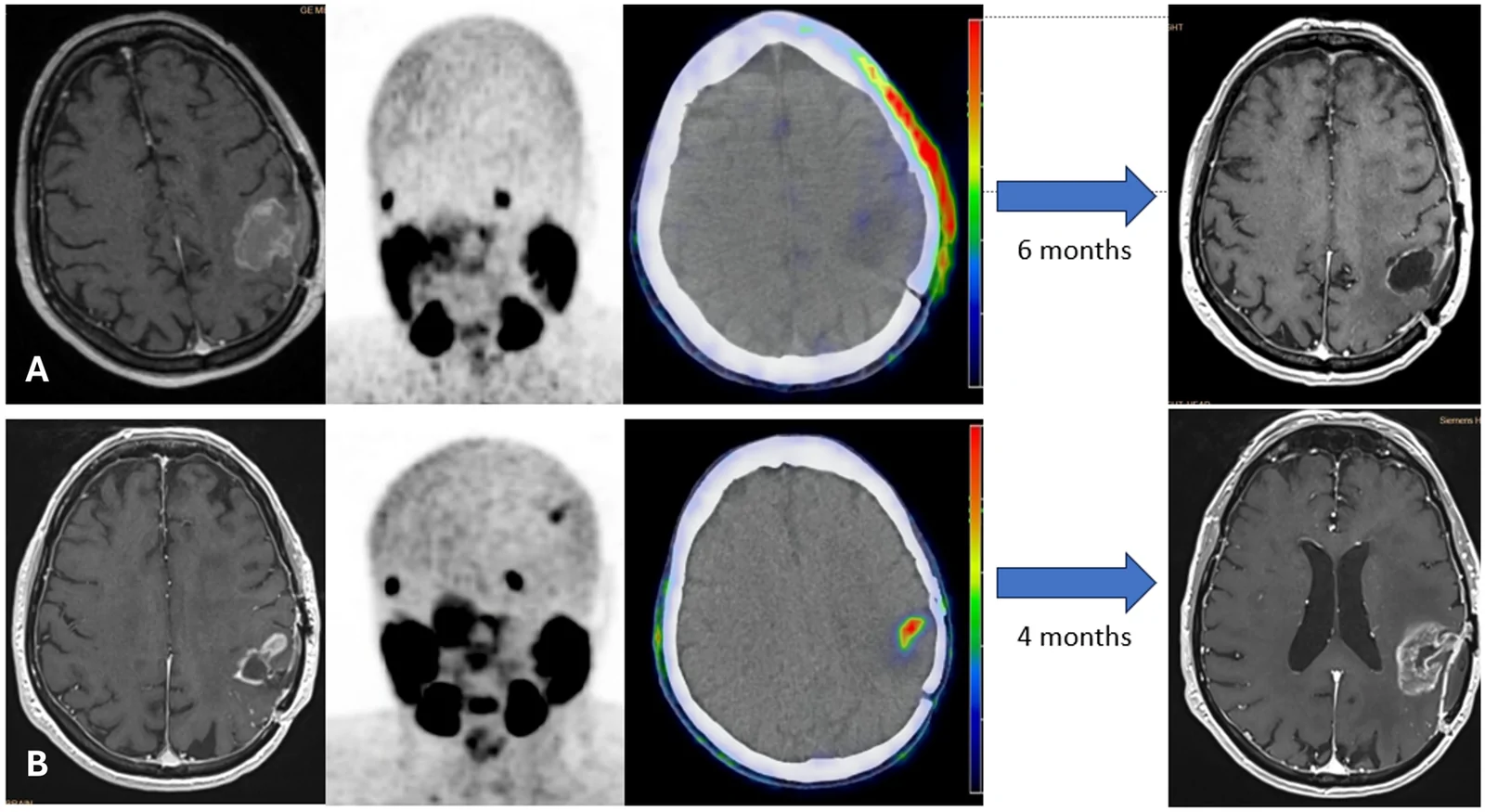
A 70-year-old gentleman with left parietal glioblastoma underwent a gross total resection (GTR), MRI (top panel) showed post-operative changes and PSMA PET CT was non-avid in the post-operative bed confirming GTR. He received 60 Gy in 15 fractions, and MRI after 6 months showed no residual disease. Unfortunately, 10 months after completing radiation, he developed failure at the edge of the cavity, as seen on the MRI (bottom panel). This was PSMA PET avid as well with a SUV of 7. He received re-irradiation 35 Gy in 10 fractions. Further imaging 4 months later showed radiation necrosis. Color Bar SUV 0-5.

**Figure 4.**
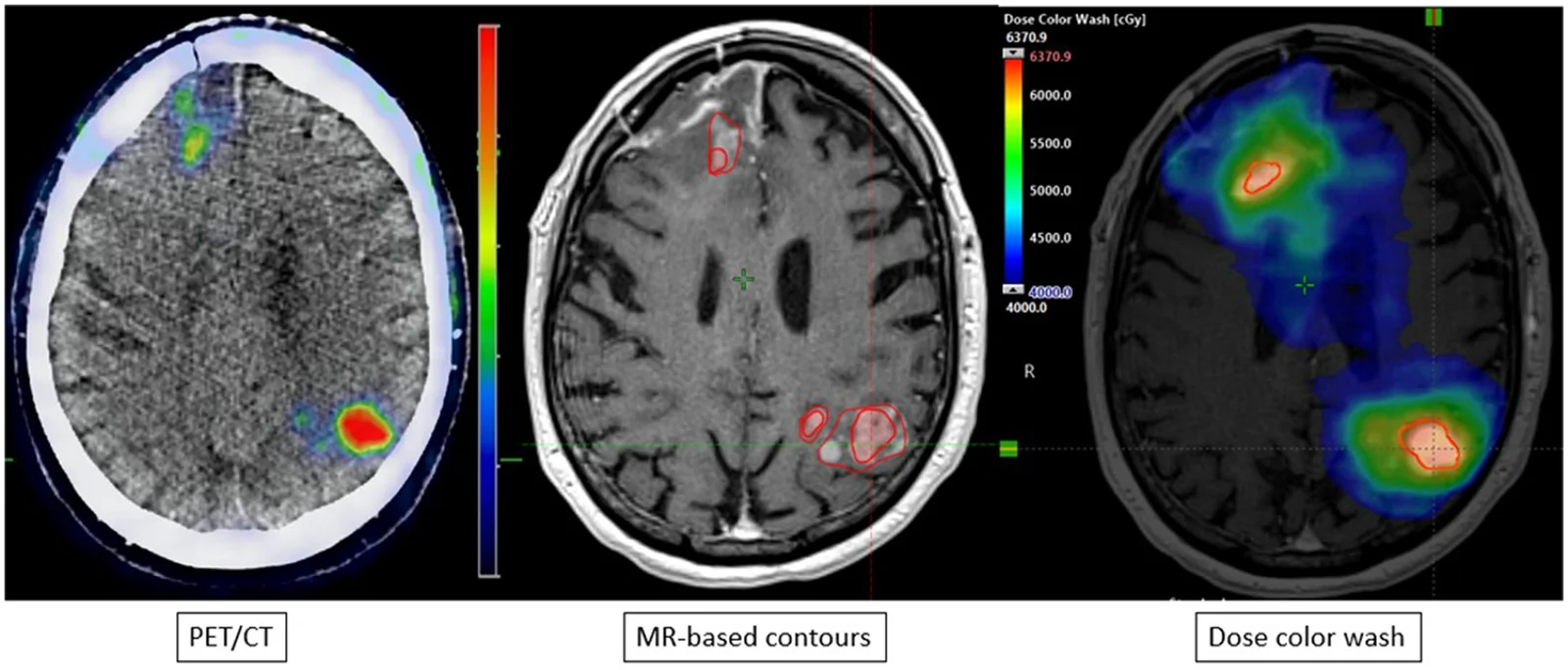
A 76-year-old gentleman presented with headaches and seizures and was found to have multifocal contrast enhancing masses in the right frontal and left parietal region. He underwent subtotal resection and pathology confirmed glioblastoma, IDH wildtype, Ki 67 30%, MGMT unmethylated. Post-operative imaging was suggestive of residual tumor, with varying degrees of PSMA PET avidity in the tumor bed. Given age and performance status, he received hypo-fractionated RT 40 Gy in 15 fractions, with a simultaneously integrated boost to 52.5 Gy to the contrast enhancing tumor, and PET guided boost to 60 Gy to PET avid tumor, as seen on the right.

### Response Assessment: Pre RT vs Post RT

Of the 19 patients in Cohort A, 13 had post-treatment follow-up PSMA PET scans available, obtained at a median of 4 months (range: 3-9 months) after completion of RT. Among these patients, 2 had complete response, 7 had a partial response, 1 had stable disease, and 3 patients had progression. Figure 1D-F demonstrates the boxplots comparing respective SUVmax, SUVRp, and SUVRsss values before and after RT in patients with available follow-up PSMA PET scans. Mean SUVmax in this patient group decreased from 6.2 to 4.6 (*P* = .005), with an average decrease of 44% in responders (range 7%-00%). Of the 2 patients with no PSMA avidity at baseline, 1 continued to be disease free (SUVmax 1.28 vs 0.6), while the other patient had a local recurrence at 10 months reflected as an increase in PSMA PET SUVmax from 1.83 to 5.71 (Figure 3, lower panel).

### Survival Outcomes

At last follow-up, 6 patients had disease progression while 7 of 19 patients had died. The median PFS from diagnosis was 10.0 months (95% CI, 6.4-13.5 months), while median OS from diagnosis was 11.1 months (95% CI, 5.5-16.7 months). Supplementary Figure S1 demonstrates the Kaplan-Meier survival curve from diagnosis. Median OS from RT was 10.1 months (95% CI, 3.6-16.7 months) with 1y-OS of 31.9%. All patients had in-field progression, including at least 1 patient with a clear progression in an area of early PET avidity (Figure 3, bottom panel).

We performed univariate analysis of factors affecting PFS and OS, including age at diagnosis, gender, extent of tumor resection, grade, IDH-status, MGMT-status, radiation dose, baseline SUVmax, SUVmean, SUVRrp, SUVRsss, and change in SUVmax and SUVmean (in patients with follow-up PSMA PET available). Increasing age was a negative prognostic factor for progression, while no other factors were significantly correlated with overall survival. Results are demonstrated in Table 2.

**Table 2.** Univariate analysis of factors affecting progression free survival (PFS) and overall survival (OS)

	PFS			OS		
Prognostic Factor	HR	95% CI	*P*-value	HR	95% CI	*P*-value
Age	1.08	1.00-1.16	.039*	1.07	0.99-1.15	.097
Gender	0.92	0.18-4.61	.92	0.91	0.17-4.80	.91
GTR	1.02	0.24-4.30	.98	0.60	0.12-3.13	.55
Grade	0.57	0.07-4.81	.60	22.8	0.00-165.55	.65
IDH-status	0.58	0.07-4.87	.62	0.04	0.00-698.52	.51
MGMT	0.13	0.01-1.08	.06	0.23	0.03-1.93	.18
RT dose	0.99	0.93-1.06	.83	0.99	0.92-1.06	.72
Baseline SUVmax	0.91	0.72-1.15	.43	1.01	0.77-1.33	.92
SUVmean	0.88	0.64-1.21	.43	0.99	0.69-1.42	.96
SUVRrp	0.34	0.01-8.40	.51	2.45	0.07-81.26	.62
SUVRsss	0.88	0.67-1.15	.34	1.04	0.83-1.31	.72
Delta SUVmax	1.27	0.60-2.70	.53	0.43	0.05-3.72	.44
Delta SUVmean	1.50	0.50-4.52	.48	0.29	0.01-5.57	.41

### Cohort-B

Eight patients underwent PSMA PET imaging at follow-up after initial treatment for suspected GBM recurrence, at a median of 8.5 months (range 1.9-13.3 months) after completing RT. Of these, 7 patients were presumed to have recurrent or residual tumors on PET, while 1 was PET negative (mean SUVmax 7.0 vs 1.5). Five of the 7 patients also had MRI findings consistent with likely tumor progression, while 2 patients had equivocal findings. The latter patient with negative PET had an indeterminate faint enhancement around the resection cavity on MRI, which initially suggested tumor recurrence (Figure 2B).

Six of the 7 patients with positive PET underwent surgery with pathology confirming progression, while 1 patient who was PET negative had pathology confirming post-treatment changes only. Mean SUVmax in patients with a positive histological correlation for progression was 7.1 (range 3.5-12.3). Among these patients, the patient with the tumor SUVmax of 3.5 had mixed tumor progression and post-treatment changes on pathological confirmation, suggesting a correlation between higher SUVmax and tumor. One patient with SUVmax of 6.8 did not have a pathological confirmation but had clear tumor progression on follow-up MRI imaging. Among 7 patients with recurrent tumors, 3 underwent re-irradiation, while 4 underwent systemic therapy alone. Two patients had a follow-up post-treatment PSMA PET with a fall in SUVmax from 7.2 and 6.8 to 5.1 and 2.3, respectively.

Figure 2B illustrates a representative case where follow-up PSMA-PET suggested RN. Although MRI revealed new peri-cavitary enhancement following chemoradiation, PSMA-PET demonstrated only minimal avidity (SUVmax 1.5). Surgical re-excision confirmed the absence of tumor progression, revealing only gliosis, again supporting correlation between PSMA PET avidity and progression.

## Discussion

Our findings suggest that post-resection PSMA PET in HGG may improve delineation of residual tumor, enable more accurate radiation treatment planning, and aid in distinguishing true tumor progression from post-treatment changes. Increased PSMA avidity quantified as mean tumor SUVmax, SUVRp, and SUVRsss was seen on PSMA PET in HGG patients with residual disease identified on postop imaging performed prior to adjuvant RT. PSMA PET showed additional areas of tumor beyond MRI-defined tumor in 20% of patients, aiding in adequate coverage, and helped in PET-guided dose escalation in 10% of patients. PSMA PET imaging was also able to accurately identify tumor progression or necrosis, with pathological correlation in 88% patients.

The lack of accurate noninvasive imaging biomarkers both at initial diagnosis and at the time of suspected recurrence poses a major obstacle in GBM management. Our approach focuses on 68Ga-PSMA, an FDA-approved PET radiotracer, which is established to have excellent clinical utility for prostate cancers but is currently not widely utilized in HGG. Our objective was to evaluate the clinical utility of PSMA-targeted PET in HGG, including GBM, to improve detection and delineation of residual tumor, with the goal of improving radiotherapy planning and response assessment. With personalized radiation treatment planning, there is potential for improving treatment outcomes in this patient population. The median overall survival from diagnosis, limited due to short follow-up, was similar to other contemporary trials.^2^^,^^3^

We observed a high tumor-to-background brain ratio (TBR) on PSMA PET imaging, which can help differentiate viable tumor from post-surgical or post-radiation changes. Similar to our results, prior studies have also shown heterogenous moderate to high PSMA avidity in de novo as well as recurrent HGG tumors on PET imaging.^18^^,^^19^ The biological substrate of PSMA uptake and the specificity of PSMA expression in HGG is an area of active investigation.^12^ Several recent in situ studies using IHC have suggested that PSMA expression is predominantly localized to the neovascular endothelium of HGG rather than the glioma tumor cells themselves.^20^^,^^21^ In another study, IHC revealed that PSMA was highly expressed in the vascular endothelium of high-grade gliomas (GBM 98%, Grade 3 gliomas 67%), whereas PSMA was poorly expressed in the vascular endothelium of RN (0%) and other brain tumors.^11^ Furthermore, recent evidence also suggests a lack of direct correlation between tracer uptake on imaging and IHC expression, postulating that blood-brain barrier (BBB) disruption may significantly contribute to the observed high PET signal.^19^^,^^22^ While PSMA PET offers a high tumor-to-background contrast, the signal is likely a composite marker of neoangiogenesis and BBB permeability rather than a specific measure of tumor cell burden.

Although it remains unclear whether PSMA-targeting tracer uptake on PET is based on PSMA-specific binding to neovasculature or nonspecific uptake in tumor, however, high TBR in HGG combined with no physiological PSMA avidity in normal healthy brain tissue (in contrast to FDG-PET) as well as in post-treatment setting can be utilized clinically to improve diagnosis and guide treatment strategies. While post-treatment changes are often challenging to differentiate from viable residual tumor on MRI, PSMA PET can provide complementary insights in RT planning in tumor recurrence setting.^23^

Another key finding of this study is that it establishes the significantly elevated PSMA uptake observed in residual viable HGG tumor tissue, suggesting that PSMA PET may serve as a potential biomarker for early response assessment and prognostication. In addition to assessing the treatment response by measurable decrease in size of tumor, decrease in PSMA avidity may provide information about treatment responsiveness of tumor and thus, can help in prognostication. Our results demonstrate a significant decrease in the mean tumor SUVmax post-RT as compared to baseline pre-RT PSMA PET imaging. The decrease in PSMA avidity probably correlates to reduced neoangiogenesis, as shown in some prospective studies with histopathological correlation.^12^ PSMA PET can also help in early identification of patients with true progression, one such case is shown in Figure 4. As further detailed below in comparison between PSMA and amino acid PET, PSMA PET can potentially identify areas of neoangiogenesis early in tumor regions that are not overly metabolic yet helping in early diagnosis of recurrent tumor.

An important application for PSMA PET/CT in HGG is in the setting of suspected tumor recurrence. Given the overlapping MR imaging characteristics of RN and tumor progression, additional imaging techniques are warranted to carefully select patients who may benefit from surgical interventions, re-irradiation, or other treatment options. In our study, PET avidity was highly correlated with tumor recurrence. As shown in Figure 2B, a low PSMA PET SUVmax of 1.5 identified RN confirmed on pathology, while higher SUV >3.5 was correlated with tumor progression.

Several amino acid PET tracers, such as 11C-Methionine (MET) and 18F-Fluoroethyltyrosine (FET), have been utilized to differentiate pseudoprogression from viable tumor in glioblastoma.^24-26^ However, these are not yet FDA-approved for use in the Unites States and are not widely available for research studies either.^27^ Vascular PSMA expression in gliomas has been shown to be a high-grade specific feature associated with elevated amino acid metabolism and short survival.^28^ In a study by Pessina et al evaluating MET PET for newly diagnosed GBM, MET uptake changed RT volume in 11% of patients, and the presence of MET uptake in patients receiving MRI-defined GTR significantly influenced survival.^26^ Studies comparing PSMA and amino acid PET such as MET PET and FET PET have shown that while amino acid PET is superior for defining the infiltrative “low-grade” edge of a tumor, PSMA PET is a more specific marker for the “high-grade” angiogenic core. Comparison between PSMA and MET-PET for radiation planning in recurrent GBM showed an increase in tumor volume based on PSMA PET, while MET-based volume was very similar to MRI-based volume, highlighting different mechanisms of PET metabolic activity.^29^ In another study that compared 68Ga-PSMA-617 and FET PET for recurrent GBMs, PSMA-avid tumor volume was larger than the FET tumor volume (*P* = .063), and PSMA had significantly higher TBR than FET (*P* = .002).^23^ Overall, accumulation of PSMA beyond FET-avid tumor regions suggests the presence of neoangiogenesis in tumor regions that are not overly metabolically active yet. Higher TBR and targeting larger tumor volume at sites of neoangiogenesis suggests that PSMA could be a better imaging biomarker, especially for recurrent tumor delineation and treatment planning than amino acid PET and MRI alone.

Another additional advantage of PSMA PET compared to amino acid tracers in this setting is the potential for theranostics with 177Lutetium (177Lu)-PSMA or 225Actinium (225Ac)-PSMA.^30^ Despite recent advances such as systemic therapies and tumor-treating fields, outcomes for patients with GBM remain poor, underscoring the need to investigate novel therapeutic strategies for this population. PSMA PET-directed therapies can be used for “relative” dose escalation to the viable and active tumor. Studies have demonstrated high PSMA uptake in HGG, including GBM, after intravenous infusion of 177Lu-PSMA.^31^^,^^32^ Several ongoing/planned prospective studies are evaluating 177-Lutetium therapy for GBMs, including 177Lu-NeoB study (NCT05739942) and FDOPA and PSMA PET for GBM in France (NCT03903419). In addition, there could be a further role of the alpha-emitter Actinium-PSMA, which has a higher linear energy transfer and help in relative dose escalation for these radio-resistant tumors.

Despite the promising feasibility of 68Ga-PSMA PET/CT in HGG, there are several limitations to our study that must be acknowledged. First, the sample size is small and heterogeneous, encompassing both treatment-naïve and recurrent cases, which limits the statistical power to draw definitive conclusions regarding diagnostic accuracy. Second, the lack of systematic histopathological validation means that the spatial correlation between PSMA avidity and viable tumor tissue remains inferred; future studies incorporating histopathological findings are required to confirm these findings. In addition, the biological specificity of the PSMA signal remains a challenge—as the PSMA expression is primarily localized to the neovascular endothelium, the PET signal may be influenced by nonspecific factors such as blood-brain barrier (BBB) disruption or inflammatory changes post-RT, potentially complicating the differentiation of tumor progression from pseudo-progression. Finally, the non-randomized design and the lack of a head-to-head comparison with established amino acid tracers such as FET and MET limit our ability to define the clinical superiority of PSMA PET. Nevertheless, our results are hypothesis-generating, suggesting the feasibility of PSMA PET in this setting, and highlight the need for larger, multi-center prospective studies to validate these results and explore the use of radiopharmaceuticals in high-grade gliomas.

## Conclusions

We present here a feasibility study evaluating the role of PSMA PET CT in treatment planning, response assessment, and differentiating tumor progression from post-treatment changes in patients with HGG. In conclusion, HGG, including GBM, demonstrates significant uptake of PSMA tracer, reflecting elevated PSMA expression in tumor microvasculature. PSMA avidity beyond conventional imaging-defined tumor regions suggests the presence of early neoangiogenesis in tumor regions that are not overly metabolically active yet, and hence PSMA could be a better imaging biomarker for early tumor recurrence. This, along with high TBR, facilitates improved tumor detection and delineation, which may enhance radiation treatment planning and response assessment. The integration of PSMA PET/CT into treatment planning for high-grade gliomas holds promise for revolutionizing the management of these tumors, with a potential for radiopharmaceutical targeting. Further clinical research is warranted to identify patients who may benefit most from PSMA PET-guided imaging for treatment planning and response assessment and facilitate the development of targeted theranostic approaches.

## Supplementary Material

vdag217_Supplementary_Data

## Data Availability

Research data are stored in an institutional repository and will be shared upon request to the corresponding author.
